# Effective reinforcement learning following cerebellar damage requires a balance between exploration and motor noise

**DOI:** 10.1093/brain/awv329

**Published:** 2015-12-01

**Authors:** Amanda S. Therrien, Daniel M. Wolpert, Amy J. Bastian

**Affiliations:** ^1^ 1 Kennedy Krieger Institute, Center for Movement Studies, 707 N Broadway, Baltimore, MD, USA; ^2^ 2 Johns Hopkins University School of Medicine, Department of Neuroscience, 725 N Wolfe St., Baltimore, MD, USA; ^3^ 3 University of Cambridge, Department of Engineering, Trumpington St. Cambridge, UK CB2 1PZ, UK

**Keywords:** reinforcement learning, adaptation, visuomotor rotation, ataxia, cerebellum

## Abstract

**
See Miall and Galea (doi: 10.1093/awv343 ) for a scientific commentary on this article.
**

Reinforcement and error-based processes are essential for motor learning, with the cerebellum thought to be required only for the error-based mechanism. Here we examined learning and retention of a reaching skill under both processes. Control subjects learned similarly from reinforcement and error-based feedback, but showed much better retention under reinforcement. To apply reinforcement to cerebellar patients, we developed a closed-loop reinforcement schedule in which task difficulty was controlled based on recent performance. This schedule produced substantial learning in cerebellar patients and controls. Cerebellar patients varied in their learning under reinforcement but fully retained what was learned. In contrast, they showed complete lack of retention in error-based learning. We developed a mechanistic model of the reinforcement task and found that learning depended on a balance between exploration variability and motor noise. While the cerebellar and control groups had similar exploration variability, the patients had greater motor noise and hence learned less. Our results suggest that cerebellar damage indirectly impairs reinforcement learning by increasing motor noise, but does not interfere with the reinforcement mechanism itself. Therefore, reinforcement can be used to learn and retain novel skills, but optimal reinforcement learning requires a balance between exploration variability and motor noise.

## Teaser

Cerebellar lesions impair both coordination and motor learning. Therrien *et al.* show that affected individuals can learn using a reinforcement mechanism despite a deficit in error-based motor learning. They also identify a critical feature of cerebellar patients’ movements (motor noise), which determines the effectiveness of learning under reinforcement.

## Graphical abstract

**Figure:**
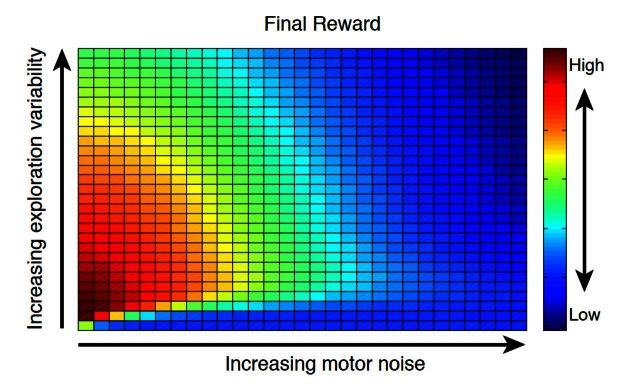
**
See Miall and Galea (doi: 10.1093/awv343 ) for a scientific commentary on this article.
** Cerebellar lesions impair both coordination and motor learning. Therrien *et al.* show that affected individuals can learn using a reinforcement mechanism despite a deficit in error-based motor learning. They also identify a critical feature of cerebellar patients’ movements (motor noise), which determines the effectiveness of learning under reinforcement.

## Introduction

Motor learning relies on interacting mechanisms that are thought to engage different neural circuits (for recent reviews see Haith *et al.* , 2013 ; Taylor and Ivory, 2014 ). Two major classes of motor learning are supervised and reinforcement learning, which have typically been studied in isolation. The extent to which these processes interact both behaviourally and neurally is not understood.

The form of supervised learning that has been studied most extensively is error-based adaptation. This mechanism requires subjects to have access to the error arising from their action—in a reaching movement this might be the vector from their final hand position to a target. Error-based learning is typically driven by predictable perturbations to a movement, resulting in errors that are corrected on a trial-by-trial basis ( Helmholtz, 1962 ; Shadmehr and Mussa-Ivaldi, 1994 ; Krakauer *et al.* , 2000 ). Many studies have shown that people with focal cerebellar damage have deficits in error-based learning of visuomotor ( Weiner *et al.* , 1983 ; Martin *et al.* , 1996 ; Tseng *et al.* , 2007 ) and force field perturbations in reaching movements ( Mashke *et al.* , 2004 ; Smith and Shadmehr, 2005 ) as well as split-belt adaptation in walking ( Morton and Bastian, 2006 ). Loss of this adaptive ability results in poorly calibrated movements.

In contrast, reinforcement learning relies on simple scalar measures of outcome such as success or failure. This mechanism requires subjects to explore in order to determine which actions will lead to success and those movements are reinforced. Learning using this mechanism can be driven by reward prediction errors, allowing one to select advantageous actions based on the probability of their yielding future rewards (for review see Sutton and Barto, 1998 ; Lee *et al.* , 2012 ). Indeed, most real-world tasks, from learning to walk to causing a swing to go higher, do not have an explicit error at each point in time. Therefore, such tasks rely on reinforcement signals (e.g. the height of the swing). Simple laboratory examples of reinforcement learning involve rewarding reaches that conform to some hidden feature the experimenter wishes the participant to learn, such as the curvature of a movement ( Dam and Kӧrding, 2013 ; Wu *et al.* , 2014 ) or an unseen visuomotor rotation ( Izawa and Shadmehr, 2011 ).

Reinforcement learning has been thought to function independently of cerebellar processes, instead relying on circuits involving the basal ganglia (for review see Knowlton *et al.* , 1996 ; Schultz, 2006 ; Ramayya *et al.* , 2014 ). In addition there are behavioural differences. For example, when learning a visuomotor rotation, reinforcement does not lead to recalibration of proprioception, unlike error-based learning ( Izawa and Shadmehr, 2011 ). Thus, reinforcement learning may allow individuals to update their movements without relying on sensory prediction mechanisms. Izawa and Shadmehr (2011) hypothesized that reinforcement learning may represent a spared mechanism for motor learning following cerebellar damage, but this has yet to be formally studied.

Here we examine how learning a new reaching movement differs in acquisition and retention between conditions with error-based versus binary reinforcement feedback. By comparing a gradual error-based adaptation and two binary reinforcement tasks with different reward schedules, we show that reinforcement learning enhances retention compared to adaptation. Examining cerebellar subjects in both error-based and binary reinforcement tasks we found that they show both learning and retention in the reinforcement task, although less than age-matched controls. Using a mechanistic model we demonstrate that the deficit appears not to be in the reinforcement learning algorithm itself, but is attributable to additional motor variability, which reduces the efficacy of reinforcement learning.

## Materials and methods

### Subjects

The study was approved by the Johns Hopkins institutional ethics review board and all subjects gave written informed consent prior to participation. For Experiment 1, 60 right-handed young controls were recruited and were divided into three groups (six males and 14 females each), each performing one of the experimental tasks: error-based adaptation (error-based group: 26.2 ± 11.1 years), open-loop reinforcement (open-loop group: 22.0 ± 3.1 years), closed-loop reinforcement (closed-loop group: 25.2 ± 5.4 years). For Experiment 2, we recruited 12 patients with cerebellar degeneration and 12 healthy controls matched for age (cerebellar group: 61.5 ± 10.0 years, control group, 59.6 ± 9.0 years) and gender (eight males, four females). Further details about the patients’ characteristics and other participants are shown in Table 1 . The severity of patients’ movement impairment was assessed using the International Cooperative Ataxia Rating Scale (ICARS; Trouillas *et al.* , 1997 ).

**Table 1:** Subject demographics

					ICARS	
Subjects	Age (years)	Sex	Handedness	Diagnosis	Total (/100)	Kinetic (/52)
CB01*	54	F	R	OPCA	36	16
CB02	51	M	R	Sporadic	64	36
CB03	63	M	R	ADCA III	12	1
CB04	61	F	R	SCA 6	55	21
CB05	42	M	L	SCA 8	59	23
CB06	61	M	R	SCA 6/8	66	25
CB07	66	F	R	ADCA III	54	18
CB08	80	M	R	ADCA III	45	23
CB09	74	M	R	Sporadic	34	8
CB10	57	M	R	SCA 7	54	49
CB11	64	M	L	SCA 6	13	4
CB12	65	F	R	SCA 6	39	19
CB group	61.5 ± 10.0	M = 8/12	R = 10/12		44.3 ± 18.1	20.3 ± 13.2
OC group	59.6 ± 9.0	M = 8/12	R = 12/12			
ERR YC group	26.1 ± 11.1	M = 6/20	R = 20/20			
OLR YC group	22.0 ± 3.1	M = 6/20	R = 20/20			
CLR YC group	25.2 ± 5.4	M = 6/20	R = 20/20			

ICARS = International Cooperative Ataxia Rating Scale; CB = cerebellar patient; OC = older control, age matched to cerebellar group; YC = young control; ERR = error-based adaptation; OLR = open-loop reinforcement; CLR = closed-loop reinforcement; F = female; M = male; R = right; L = left; OPCA = olivopontocerebellar atrophy; ADCA III = autosomal dominant cerebellar ataxia type 3 which has only cerebellar signs; SCA = spinocerebellar ataxia types 6, 7 and 8; Sporadic = sporadic adult-onset cerebellar ataxia; Group data: mean ± SD. *Subject did not complete ERR task. None of the patients or controls had sensory loss in clinical tests of proprioception and monofilament testing for tactile sensation ( Campbell, 2005 ). One patient (Patient CB01) had atrophy in the brainstem by MRI, but showed no extra-cerebellar signs. One patient (Patient CB10) presented with hyperactive reflexes in the lower limbs, but reflexes were normal bilaterally in the upper limbs. No signs of white-matter damage or spontaneous nystagmus were seen among any patients.

### Apparatus

Participants made reaching movements in a KINARM exoskeleton robot (B-KIN Technologies). Each participant’s right arm rested in trays in this device and they performed movements in the horizontal plane below a screen that prevented them from viewing their arm. All visual feedback was projected on to the screen’s surface.

### Procedure

#### Experiment 1

**Figure 1:**
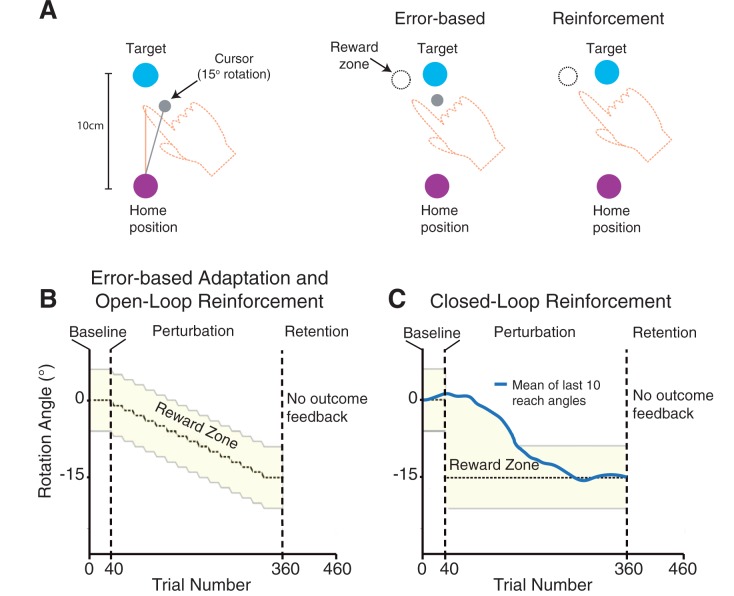
**Task overview and schematic of feedback conditions.** ( **A** ) Participants were required to make a 10 cm reaching movement from a home position to move a cursor through a visual target. The hand was hidden from view below a screen. At baseline, the visual cursor represented the index fingertip position. In the perturbation phase, the cursor was gradually rotated relative to the index fingertip. This required participants to learn to alter their reach angle to counter the rotation and move the cursor toward the visual target. Participants were given either error-based or binary reinforcement feedback. In the error-based task, the cursor was presented throughout each reach and successful reaches were those in which the cursor was seen to hit the visual target. This required participants to reach through the reward zone. In the reinforcement tasks, the cursor was not shown and participants received only binary feedback about task success or failure. Here, reaches that successfully crossed the reward zone were rewarded with the visual target turning green. If a reach failed to counter the rotation, the visual target disappeared to signal the end of that trial. ( **B** ) In the error based adaptation and the open-loop reinforcement tasks, the rotation was gradually introduced during the perturbation phase. The yellow region shows the reward zone. ( **C** ) The closed-loop reinforcement task rewarded reaches that fell between the mean of the participant’s previous 10 reaches and the outer bound of the reward zone.

Participants completed one of three reach learning tasks: an error-based adaptation, an open-loop reinforcement, or a closed-loop reinforcement task. In all tasks, we introduced a 15° visuomotor rotation between the index finger location and the position of a cursor, which participants were told represented the finger position ( Fig. 1 A). This cursor was either visible (error-based feedback) or invisible (reinforcement-based feedback) throughout each reach. Within each group the rotation direction was counterbalanced across subjects (clockwise or counter-clockwise). All participants performed a 40 trial baseline phase with no rotation, a 320 trial perturbation phase in which a visuomotor rotation was introduced and finally a 100 trial retention phase in which no visual feedback or knowledge of outcome was provided.

A trial began with the participant’s finger at the home position (1-cm radius circle) ∼40 cm directly in front the subject. Once the hand was held in the start position for 500 ms, a target appeared on the screen (1-cm radius circle). The centre of the target was positioned directly in front of the subject, 10 cm distal to the home position. Participants were asked to reach so that their index finger passed through the target. The trial ended when the hand exceeded a distance of 10 cm from the home position. Participants were asked to then relax their arm and the robot passively brought their index finger back to within 2 cm of the home position. A cursor representing the position of the index finger was then presented to allow participants to move their hand into the home position to start the next trial. To encourage participants to make movements within 200–600 ms after leaving the home position, the target turned blue or red for movements that were too slow or too fast, respectively. Prior to beginning the experiment, all participants were given a 40-trial practice block with no cursor rotation to familiarize themselves with the task and movement speed requirements.

In the error-based adaptation task, participants received cursor feedback throughout the reach (0.5-cm radius filled grey circle updated at the screen refresh rate of 60 Hz). During the perturbation phase of the task, a 15° visuomotor rotation was gradually introduced in steps of 1° every 20 trials with an additional 20 trials at the full rotation ( Fig. 1 B). Successful trials were those in which the visual cursor was seen to make contact with the visual target. When the visuomotor rotation was introduced, this required the subjects to move their finger though a rotated reward zone (dotted circle, Fig. 1 A—invisible to the participant), which had a width of ± 5.7° around the rotation angle, corresponding to the width of visual target. Thus, participants had to learn to reach at an angle, relative to the visual target, that countered the visuomotor rotation. The reach angle was defined as the angle between two vectors: one from the initial finger position to the target and the other from initial finger position to the finger location when 10 cm from the home position: movement endpoint. In this task, cursor feedback was extinguished as soon as participants’ hands crossed a 10-cm radius around the centre of the home target and was not displayed during the return of the hand to the home position.

In the two reinforcement learning tasks, participants received only binary (success or failure) feedback about the outcome of their movement—no cursor feedback was shown. If the invisible cursor, would have touched the visual target (i.e. if the finger passed through the reward zone; Fig. 1 A), the visual target turned green indicating success, otherwise the visual target disappeared to signal trial completion and that the target had been missed. In the open-loop reinforcement task, the perturbation phase introduced a gradual visuomotor rotation that was identical to the error-based task, with the exception that no cursor feedback was provided ( Fig. 1 B). In the perturbation phase of closed-loop reinforcement task, the rotation angle depended on the participant’s previous reaches. That is, the rotation angle was calculated as the moving average of participants’ previous 10 reach angles (or 15° if the moving average was greater than this; Fig. 1 C). Stated a different way, subjects were rewarded if their reach was rotated beyond the average of their last 10 reaches in the direction that countered the rotation. We only provided participants with outcome (reinforcement) feedback on valid trials; that is, trials in which the duration criteria (200–600 ms) were met. To control for possible different numbers of valid trials between participants we performed additional analyses to ensure that the number of valid trials did not affect our results.

For all tasks the retention phase was identical. All feedback was extinguished (no cursor displayed or success feedback) and participants were instructed to continue to reach and aim for the visual target.

#### Experiment 2

The objective of this experiment was to compare reinforcement learning and gradual error-based adaptation in individuals with cerebellar degeneration and healthy age-matched controls. In two sessions, performed on separate days, participants completed the error-based and closed-loop reinforcement tasks with the order counterbalanced within each group. We chose to only use a clockwise rotation in this experiment because cerebellar patients exhibit a clockwise bias in their reaches to the target we used ( Gibo *et al.* , 2013 ). By using a clockwise rotation, we ensured the participants would not perform well under the rotation simply because of their bias, and would be required to learn a new reach angle to achieve task success. Again, we only gave binary feedback in the reinforcement condition when trials met the movement speed criterion. This was particularly important for the cerebellar patients because fast movements will exacerbate their reach errors. Additional analyses were performed to control for any differences in the number of valid trials in the reinforcement condition. We chose not to add in extra trials for any invalid trials to avoid effects of fatigue.

### Measurements and analysis

Data from the clockwise and counter-clockwise conditions in Experiment 1 were analysed together—we flipped the counter-clockwise data to correspond to clockwise rotation participants. Statistical analysis used a mixed model design. In Experiment 1, between groups comparisons were made using the factor of task (Error-based, Open-loop, Closed-loop). Within groups, measures were compared over five experiment phases: the mean of the Baseline block, Early and Late Perturbation (trials 41–80 and last 40 trials), Early and Late Retention (first and last 40 trials). One-way ANOVA was used to compare total learning (difference between Late Perturbation and Baseline), early and late retention (percentage of Early and Late Retention relative to Late Perturbation) and within subject standard deviation (SD) at baseline.

In Experiment 2, a mixed-model design was used with a between subjects factor of group (Cerebellar, Older Control) and within subjects factors of task (Error-based, Closed-loop) and phase (Baseline, Early Perturbation, Late Perturbation, Early Retention, Late Retention). Total learning and early and late retention were compared between groups and within task. In this experiment only the valid trials were included in the analysis.

All *post hoc* means comparisons were performed using Bonferroni corrections for multiple comparisons. All data were tested for normality using the Shapiro-Wilk test. Homogeneity of variance was also evaluated using Mauchly’s Test of Sphericity and Levene tests for mixed-model and one-way ANOVAs, respectively. Tests of sphericity revealed unequal variance between groups across phase in Experiments 1 and 2. Levene tests also revealed unequal variance between groups for mean values for total learning, late retention and Baseline within subject standard deviation in Experiment 1 and model parameters in the modelling analysis. In these cases, Greenhouse-Geisser corrections and Brown-Forsythe tests for equality of means were used for mixed model ANOVA and one-way ANOVA, respectively, to ensure that significant effects were robust to heteroscedasticity. All data analysis was performed using custom written software in Matlab (Mathworks). In all tasks, bivariate correlations were performed between dependent variables in the cerebellar group and ICARS total scores as well as Kinetic subscores. Statistical analysis was performed using SPSS software (IBM).

To examine learning in Experiment 2 corrected for the number of feedback (valid) trials experienced, we performed a bootstrap analysis between the groups. To compare the cerebellar participants to the older group, we considered each cerebellar participant individually. First we took the difference between their reach angle, averaged over their last 20 valid trials in the perturbation session, and the average of the trials matched for the number of valid trials from a randomly selected older subject who had at least the same number of valid trials. This compares learning for the trials that occur after the same number of perturbation trials with outcome feedback. We then summed these differences across cerebellar subjects to produce a single bootstrap sample and repeated this procedure 10 000 times and calculated the *P* -value as the proportion of the samples that were less than zero. We repeated this process for the other group comparisons and for both reach angle and total learning.

### Model analysis

To model the reinforcement learning task we used a simple mechanistic model which incorporates both exploration and motor noise. We assume that on any trial, *t* , subjects have an internal estimate of the state of the rotation angle, *x*_t_ (or equivalently an estimate of the reach angle which would lead to success). We include variability in participant’s reaches from two sources, motor noise and exploration variability that both affect reach direction. The key difference between these two sources of variability is that we assume participants are unaware of the motor noise, but have full awareness of their exploration variability. On each trial, both sources of variability are modelled as draws from zero mean Gaussian distributions with standard deviations of σ _m_ and σ _e_ , for the motor and exploration components. A mathematically similar way of expressing this is that there is a total variability $\left(\sqrt{\sigma_{e}^{2}+\sigma_{m}^{2}}\right)$ in a participant’s reach direction and that he or she is aware of (or corrects for) a proportion of this variability, $\rho =\frac{\sigma_{e}^{2}}{\sigma_{e}^{2}+\sigma_{m}^{2}}$ .

On each trial the reach angle, y _t,_ is modelled as y _t_ = x _t_ + e _t_ + m _t_ , where e _t_ and m _t_ are samples of the exploration variability and motor noise, respectively. If a subject is unsuccessful and misses the target, the internal estimate of reach direction is unchanged (x _t+1_ = x _t_ ), However, if the subject is successful then they update the estimate of their reach angle by the exploratory noise on that trial, x _t+1_ = x _t_ + e _t_ . Although this formulation appears to have a learning rate of unity we can instead consider the update such that ρ is the proportion of the full variability that subjects correct for if rewarded.

To fit this stochastic model to each subject’s data we used a particle filter ( Doucet and Johansen, 2009 ). This is necessary as the model is complex as reward depends not only on the internal estimate of state of the current trial, but also on the actual reach angles on the previous 10 trials, thereby making an analytic fit intractable.

We simulated R = 10 000 particles for each setting of our model parameter $\theta =\left\{\sigma_{m},\sigma_{e}\right\}$ . We chose the parameters equally spaced on a 50 × 50 grid of σ _m_ = {0.01 − 8.5} and σ _e_ = {0.01 − 5.5} degrees. This grid was wide enough to contain the optimal estimates. For each parameter setting, all particles were initialized at the average reach angle of the Baseline phase. The particles represented estimates of the perturbation such that $x_{t}^{r}$ is that rotation estimate for particle *r* at time step *t.* Each of the T steps (corresponding to the trials) of the simulation involved:

1. Computing the weight for each particle *r*
  $$w_{t}^{r}=P\left(y_{t}|x_{t}^{r}\right)=N\left(y_{t},x_{t}^{r},\sqrt{\sigma_{m}^{2}+\sigma_{e}^{2}}\right)$$
2. Calculating an estimate of the likelihood for that data point
  $$l_{t}=\left(1/R\right)\sum_{r}w_{t}^{r}\approx P\left(y_{t}|y_{1},\ldots ,y_{t-1}\right)$$
3. Normalizing the weights so that they sum to 1 across the particles
  $${\hat{w}}_{t}^{r}=w_{t}^{r}/\sum_{r}w_{t}^{r}$$
4. Resampling R particles such that for each sample the probability of sampling particle *r* is ${\hat{w}}_{t}^{r}$ .
5. if *t* < *T* go to 1 with *t* = *t* + 1

The key idea behind the particle filter is that the set of particles represent the posterior distribution over the rotation angles and that we can calculate the log likelihood of the data given the parameters as

$$\log L(\theta )=\sum_{t}\log l_{t}$$

For each subject we found the parameters that maximized the log-likelihood. Note that unlike fitting mean square error ( Izawa and Shadmehr, 2011 ) this method takes into account the non-independence across trials of the simulated and actual data. Confidence intervals were calculated using the profile likelihood method (Appendix A in McCullagh and Nelder, 1989 ). That is, the (1 − α) confidence interval encloses all values of θ for which the log likelihood is within $\chi_{1-\alpha }^{2}(d)/2$ of the maximum log likelihood, where d is the number of parameters (2) being estimated via the method of maximum likelihood. To show sample simulation of the fits we simulated 10 000 simulations using the maximum likelihood parameters and calculated the mean squared error to the subjects’ data and chose the simulation with the median mean squared error.

To evaluate the model’s goodness of fit we compared the log-likelihood of the data (using R = 1000 particles) for each subject, using the maximum likelihood parameters of the model, to the log-likelihood of 1000 simulated data sets generated from the model with the same maximum likelihood parameters ( Goris *et al.* , 2014 ). We considered a model fit acceptable if the log-likelihood of the real data lay within the central 95% of the distribution of log-likelihood of the simulated data. This was the case for the vast majority of participants (30 of 34) suggesting the model is generally acceptable for this task.

## Results

### Behavioural analyses

#### Experiment 1

**Figure 2:**
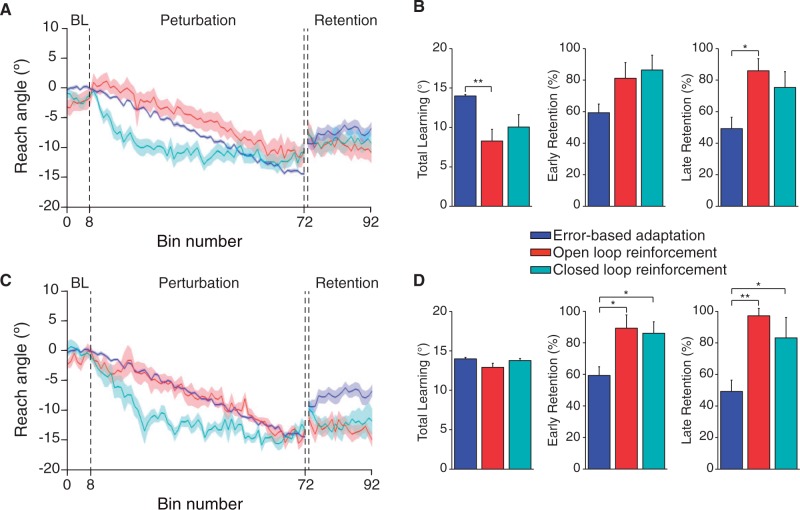
**Comparison of error-based, open- and closed-loop reinforcement.** ( **A** ) Reach angle (group mean ± SEM) across trials for the error-based adaptation, open- and closed-loop reinforcement tasks. ( **B** ) Group mean of total learning ( *left* ), early retention ( *middle* ) and late retention ( *right* ). ( **C** ) Reach angle (as in **A** ) from a subset of the subjects in each of the open- and closed-loop reinforcement groups (10 of 20) chosen to match the learning in the error-based group. ( **D** ) Group mean results for data in **C** . Error bars indicate SEM. * *P* < 0.05, ** *P* < 0.01.

The experiment was designed to compare the learning and retention of visuomotor rotations under error-based feedback and two types of reinforcement feedback. Figure 2 A shows the time series of reach angles for the different groups. All groups could reach within the target zone during the baseline phase. During the perturbation phase all groups learned and on average were within the final target zone centred on 15°. The open- and closed-loop groups were able to match the desired duration time window for most trials and did not have a significantly different proportion of trials outside the range (invalid trials, 5.7% and 3.7%, respectively, *P* = 0.18). During retention, when both the cursor and outcome feedback were removed, each group initially maintained their learned reach angle, though the error-based group showed some decay over this period.

A mixed model ANOVA of reach angle showed no group effect, but a significant effect of experimental phase [ *F* (4,228) = 79.176, *P <* 0.001; Greenhouse-Geisser corrected, *F* (2.267,129.198) = 79.176, *P <* 0.001] and a significant group × phase interaction [ *F* (2,228) = 6.849, *P <* 0.001]. Thus, the three groups responded differently across phase. We used *post hoc* comparisons to examine the key features of learning and retention. We found differences at baseline with the open-loop group having a significantly more negative reach angle compared to the error-based group ( *P <* 0.05). In addition the closed-loop group showed significantly more learning in the early perturbation phase compared to the error-based and open-loop groups (both *P <* 0.001). All groups showed significant learning from baseline to the late perturbation phase (all *P <* 0.001) and similar reach angles early in retention, but in late retention the error-based group showed significant decay from late perturbation compared to the other groups ( *P <* 0.001). This suggests that learning with error feedback yielded reduced retention of the learned movement relative to reinforcement feedback.

Figure 2 B shows total learning (difference between Baseline and End Perturbation) and the per cent early and late retention (measured as the ratio of Early and Late Retention angle to End Perturbation). One-way ANOVA on total learning revealed a significant main effect of group [ *F* (2,57) = 5.446, *P <* 0.01], driven by a significant difference between the error-based and open-loop group. This effect was preserved in Brown-Forsythe testing of mean differences, which accounts for unequal variances within groups [ *F* (2,38.439) = 5.446, *P <* 0.01].

One-way ANOVA showed similar early retention (mean of first 40 retention trials) for all groups [ *F* (2,57) = 2.833, *P =* 0.067] but a significant difference in late retention [mean of last 40 retention trials *F* (2,57) = 5.056, *P <* 0.05] and *post hoc* test showed this was driven by the decay in the error-based group compared to the open-loop group ( *P <* 0.05). To examine differences in retention between groups when equated for total learning (and therefore discounting potential effects of the number of valid trials), we selected 10 subjects (of 20) from each of the open-loop and closed-loop groups so as to match their total learning mean and variance to the error-based group. To do this we computed the mean and variance of all possible subgroups of 10 subjects in the open-loop and closed-loop groups, respectively. We chose the subgroup that minimized the Jensen-Shannon divergence between that subgroup and the error-based group’s total learning distributions. Figure 2 C shows the time series of reach angles for these matched subgroups and the error-based group. Group means for total learning were not significantly different following matching, [ *F* (2,37) = 3.215, *P =* 0.052; Brown-Forsythe, *F* (2,17.182) = 2.645, *P =* 0.10; Fig. 2 D]. Analysis of both early and late retention in the matched subgroups revealed a significant main effect of group [early retention, *F* (2,37) = 6.573, *P <* 0.01; late retention, *F* (2,37) = 8.916, *P <* 0.01; Fig. 2 D]. The main effect for late retention was preserved following Brown-Forsythe tests [ *F* (2,19.406) = 9.097, *P <* 0.01] indicating that the result was robust to unequal variance across groups. In both early and late retention, main effects were driven by significantly reduced retention in the error-based group compared with the two reinforcement-learning groups. Thus, learning with reinforcement feedback yielded enhanced retention of the learned reach angle compared with online error feedback.

To examine variability in performance we analysed within subject standard deviations in the reach angles of the overall groups over the 40 baseline trials. One-way ANOVA revealed a significant main effect of group [ *F* (2,57) = 7.251, *P <* 0.01], which was repeated following Brown-Forsythe testing [ *F* (2,42.044) = 7.251, *P <* 0.05]. *Post hoc* comparisons revealed that the effect resulted from significantly greater within subject reach angle variability in the two reinforcement groups compared to the error-based group (both *P <* 0.01). The main effect of group was replicated when within subject reach angle standard deviation was analysed in the resampled reinforcement groups matched to the performance of the error-based group [ *F* (2,37) = 7.496, *P <* 0.01; Brown-Forsythe, *F* (2,20.587) = 5.695, *P <* 0.05]. Overall, these findings suggest that online error feedback reduces behavioural variability compared with binary reinforcement feedback.

#### Experiment 2

**Figure 3:**
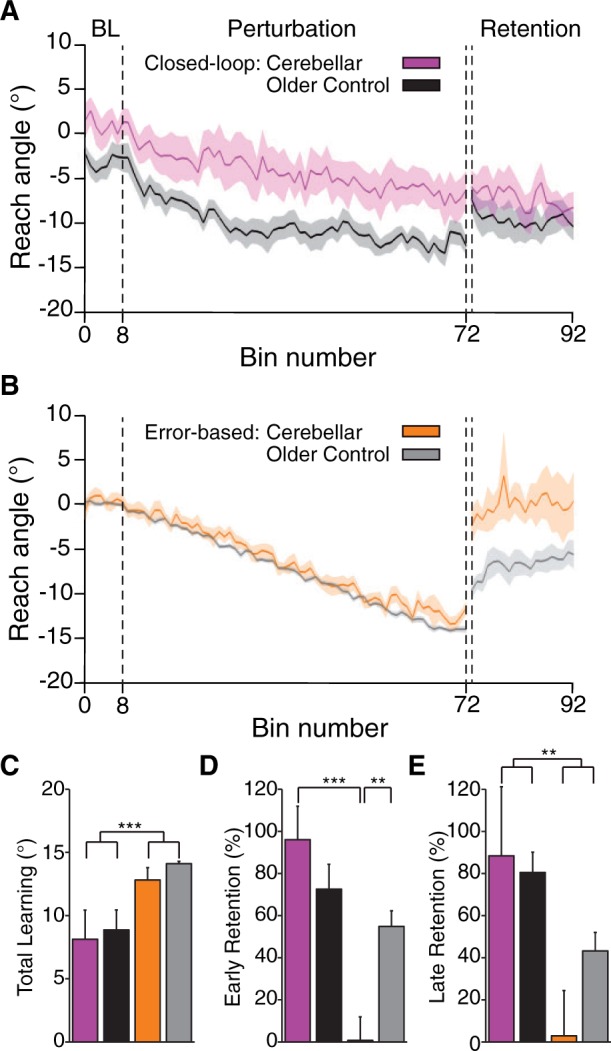
**Comparison of error-based and closed-loop reinforcement in cerebellar subjects and matched controls.** ( **A** ) Reach angle (group mean ± SEM) across trials for the closed-loop reinforcement task. On average, the cerebellar patients did not reach the final reward zone, unlike control subjects, but maintained the learned reach angle once reinforcement feedback was removed in the retention phase **B.** Reach angle (as in **A** ) for the error-based adaptation task. The cerebellar patients were able to follow the gradual visuomotor rotation (as did control subjects) but showed no retention of the rotation once feedback was removed. ( **C** ) Group mean results for total learning ( *left* ) and per cent early and late retention ( *middle* and *right* , respectively) in the closed-loop reinforcement and error-based adaptation tasks. Error bars indicate SEM. ** *P* < 0.01, *** *P* < 0.001.

This experiment was designed to compare reinforcement learning and error-based learning in individuals with cerebellar damage and age-matched, healthy controls (older controls). Both groups could, on average, reach within the target zone during the baseline phase of both tasks ( Fig. 3 A and B). By the late perturbation phase, control subjects were on average within the final target zone (centred on 15°) in both tasks. However, the cerebellar group on average only reached the final target in the error-based task. Both groups maintained their learned reach angle throughout retention in the closed-loop task. In the error-based learning task, older control subjects initially maintained their learned reach angle, but their performance decayed over the retention period. In contrast, the cerebellar subjects showed no retention as their performance immediately returned to close to baseline.

We used a mixed-model ANOVA to examine differences in reach angle between the groups, tasks and phases. We found significant main effects of group [ *F* (1,21) = 8.6, *P <* 0.01], task [ *F* (1,21) = 6.3, *P <* 0.05] and phase [ *F* (4,84) = 50.1, *P <* 0.001; Greenhouse-Geisser corrected, *F* (2.4,49.8) = 50.1, *P <* 0.001]. The interaction among these factors was also significant [ *F* (4,84) = 4.73, *P <* 0.01]. *Post hoc* analysis showed that both groups learned from baseline to the late perturbation phase in both tasks (for the cerebellar group in the closed-loop task, *P <* 0.05, all others, *P <* 0.001). Total learning was greater for the error-based task compared to the reinforcement task [ *F* (1,21) = 24.7, *P <* 0.001, Fig. 3 C]. Although there were differences in the mean learned reach angle between older control group and cerebellar group in the closed-loop task ( *P <* 0.01), total learning was not significantly different across the groups in either task ( *P =* 0.235).

In the closed-loop condition, the cerebellar and older control group did not have a significantly different proportion of invalid trials (10.9 and 11.5%, respectively, *P =* 0.90) whereas the young controls had only 1.7% invalid trials. We used a bootstrap analysis to compare reach angles at the end of the perturbation phase as well total learning, both adjusted for the number of valid trials (see ‘Materials and methods’ section). This showed that the cerebellar group had a significantly smaller reach angle at the end of the perturbation phase compared to the older and younger groups ( *P <* 0.001), but the older group was not significantly different from the younger group ( *P =* 0.24). However, for total learning all groups showed significantly different amounts of learning with the older learning more than the cerebellar group ( *P =* 0.026) and the younger learning more than the older group ( *P <* 0.001).

In the error-based task, both groups showed a significant decay from late perturbation to early and late retention (older control: *P <* 0.001, *P <* 0.05, respectively; cerebellar: both *P <* 0.001). However, the decay in early retention was much greater for the cerebellar group as they returned to near baseline performance ( *P <* 0.001).

To take into account the final learning level, we examined the per cent early and late retention ( Fig. 3 D and E). Mixed model ANOVA of per cent early retention revealed a significant main effect of task [ *F* (1,21) = 54.8, *P <* 0.001] and a significant interaction among factors group and task [ *F* (1,21) = 15.2, *P <* 0.01, Fig. 3 D]. *Post hoc* analysis showed that the cerebellar group had reduced retention in the error-based task relative to the closed-loop task ( *P <* 0.001) and reduced retention in the error-based task compared to controls ( *P <* 0.01). ANOVA of per cent late retention revealed a significant main effect of task [ *F* (1,21) = 11.3, *P <* 0.01, Fig. 3 E], which was driven by reduced retention in both groups the error-based task compared to the closed-loop task. Together these results suggest that cerebellar patients retained their learning in the closed-loop task similarly to age-matched controls. Conversely, while control participants showed some early retention of the adaptation in the error-based task, the cerebellar group showed no such retention.

**Figure 4:**
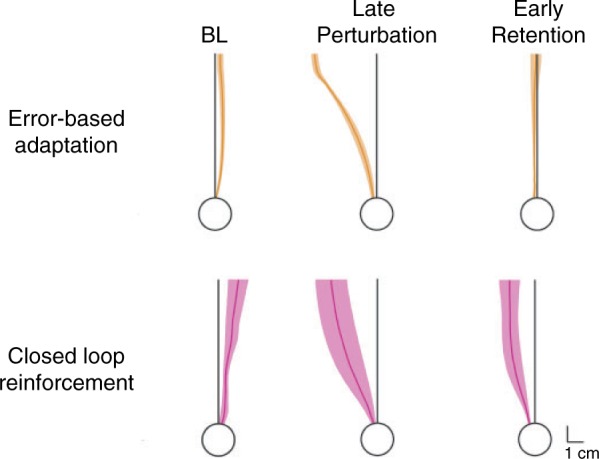
**Hand paths for the cerebellar subjects in the error-based adaptation and closed-loop reinforcement tasks.** The *left* column shows the group mean over the 40 baseline (BL) trials. The middle and right columns show late perturbation and early retention paths. Paths were first averaged within a subject and then averaged across subjects and are shown ± SEM.

We were surprised that the cerebellar group showed almost complete adaptation in the error-based task, despite no retention. This discrepancy could arise if the cerebellar group relied on online visual feedback to control their reaches. In support of this, examination of the initial portion of the trajectory in the error-based group appears more directed toward the visual location of the target late in learning ( Fig. 4 , middle) and only curves towards the target zone as the movement progresses. In contrast the initial trajectory in the closed-loop task (in which visual feedback cannot be used) is aimed towards the target zone. In early retention ( Fig. 4 , right) the error-based group make movements almost straight to the target suggesting that the curvature seen in the later perturbation movements are driven by visual feedback. In the closed-loop task, however, the cerebellar group maintains some of their initial trajectory deviation in the early retention period. This suggests that the cerebellar group used online feedback to correct their movements in the error-based task resulting in the appearance of complete adaptation, but without any true updating of feedforward models of the correct movement.

**Figure 5:**
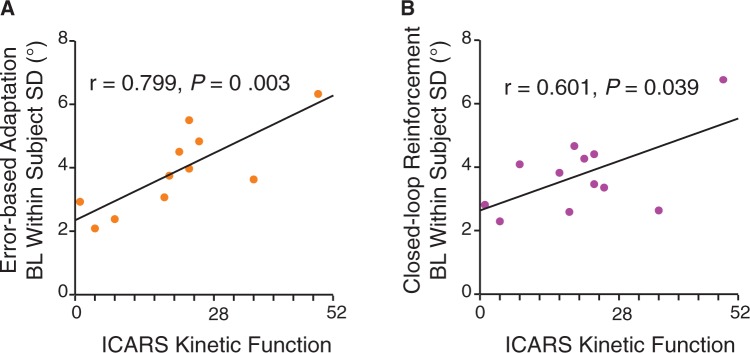
**Baseline movement variability correlates with ICARS kinetic function score.** Plots show correlations between within subject reach angle standard deviation at baseline and kinetic function subscore on the International Cooperative Ataxia Rating Scale (ICARS). ( **A** ) Error-based adaptation task. ( **B** ) Closed-loop reinforcement task.

To examine movement variability we analysed baseline within subject reach angle standard deviation for each subject in the two tasks. Mixed model ANOVA revealed a significant main effect of group that was the result of greater reach angle variability in the cerebellar group compared to controls [ *F* (1,21) = 14.0, *P <* 0.01]. The group × task interaction was also significant [ *F* (1,21) = 8.3, *P <* 0.01]. Within the older control group, reach angle variability was greater in the closed-loop task compared to the error-based task ( *P <* 0.01). Baseline reach angle standard deviation in the cerebellar group was related to their clinical impairment. The kinetic function sub-score of the International Cooperative Ataxia Rating Scale (ICARS), which is the component of the scale directly related to arm ataxia, was correlated with baseline variability in both the error-based ( *r* = 0.8, *P <* 0.01, Fig. 5 A) and closed-loop tasks ( *r* = 0.6, *P <* 0.05, Fig. 5 B). No other significant correlations were found between dependent variables and ataxia rating scores.

### Modelling

Many of the cerebellar subjects showed significant learning and retention in the reinforcement condition. Further, we saw individual differences in reinforcement learning within the cerebellar and control groups with some subjects showing substantial learning and others showing little learning. In an attempt to understand these differences, we developed a simple mechanistic model of the reinforcement learning task and fit each subject’s closed-loop data. The model considered the reach angle executed on a given trial to be the result of an internal estimate of the ideal reach angle (i.e. to counter the current rotation applied) with the addition of two sources of variability: motor noise and exploration variability. The important difference between these two sources of variability is that we assume participants are unaware of their motor noise, but have full awareness of their exploration variability. When an action is rewarded, the subject updates their internal estimate of reach angle based solely on the contribution of exploration variability. When an action is unrewarded, the reach angle is not updated. The model has two parameters, the standard deviations of the Gaussian distributions that generate the motor noise and exploration variability. We fit the model to each individual subject’s data using maximum likelihood estimation.

**Figure 6:**
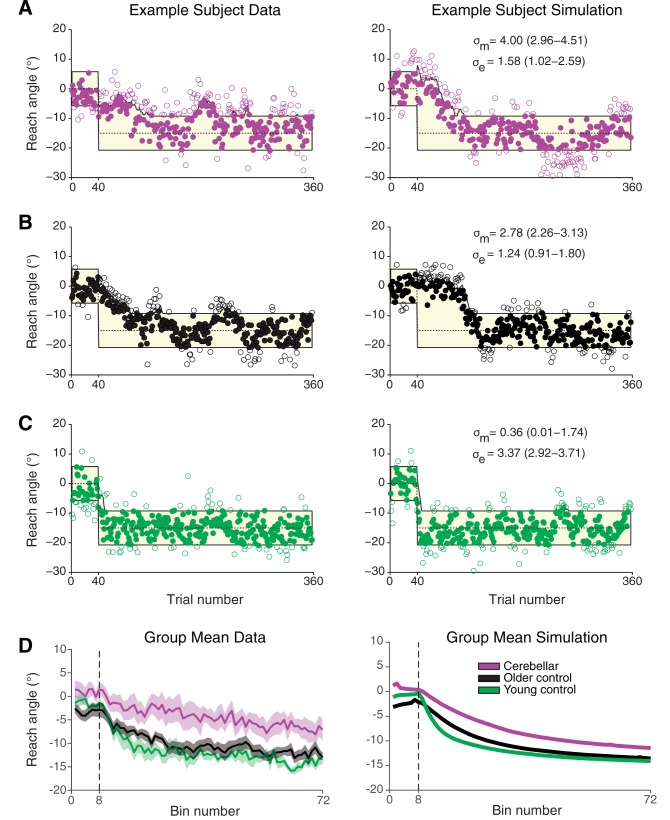
**Empirical data and model simulations for example subjects and group means.** ( **A–C** ) *Left* : Data from an example ( **A** ) cerebellar subject; ( **B** ) older control subject; and ( **C** ) young control subject. ( **A–C)***Right* : Single run simulation with the maximum likelihood parameter fits for motor noise, σ _m_ , and exploration variability, σ _e_ , for the corresponding example subjects. From the set of Monte Carlo simulations, we chose to display the simulation which had the median mean squared error between data and simulation. Parameter fits show 95% confidence intervals indicated in parentheses. The yellow region shows the reward zone and the filled symbols are trials that were rewarded. ( **D** ) Group mean reach angle ( *left* ) and mean simulation ( *right* ) for the cerebellar patient, older control and young control groups in the closed-loop reinforcement task.

Three typical subject’s data (a cerebellar, old and young control) are shown along with a typical simulation for each ( Fig. 6 A–C and parameter estimates, see ‘Materials and methods’ section). The cerebellar subject had the largest motor noise, followed by the older control with the young control having the smallest amount of motor noise. In contrast, the younger control had the largest exploration variability. These parameters led to slower learning for the cerebellar and older control and faster learning for the younger control. Figure 6 D shows that group means match reasonably well to the mean of the simulations (averages of 10 000 simulations with each subjects fitted parameter).

**Figure 7:**
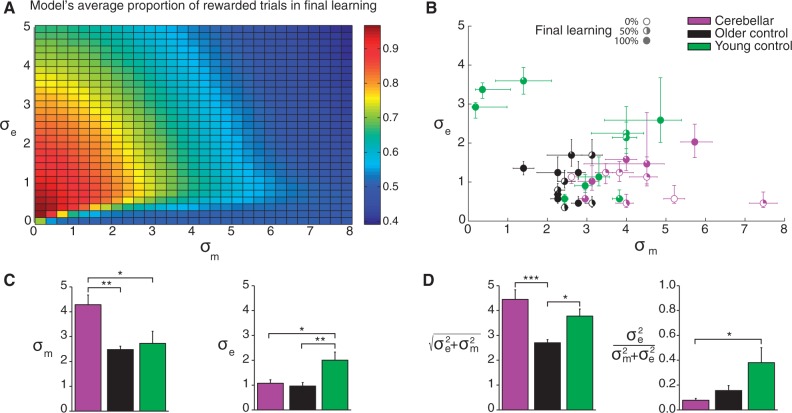
**Model analysis of the closed-loop reinforcement task in cerebellar subjects, older control subjects and young control subjects.** ( **A** ) Proportion of rewarded trials in late learning from simulations of the model with different parameter settings on a 30 × 30 grid. ( **B** ) Individual subject parameter fits with 95% confidence intervals. The per cent of final learning for each subject is indicated by the fill of each subject’s marker. In general, subjects with greater motor noise, σ _m_ , and lower exploration variability, σ _e_ , show less learning. ( **C** ) Mean model ( ± SEM) parameters across subjects for motor noise ( *left* ) and exploration variability ( *right* ). ( **D** ) Model parameters expressed in a mathematically alternative way, as total variability ( *left* ) and the proportion of total variability that the motor system adjusts for when successful ( *right* ). * *P* < 0.05, ** *P* < 0.01, *** *P* < 0.001.

The model predicts that the amount of learning and reward depends on both the motor noise and exploration variability. Figure 7 A shows the proportion of rewarded trials in late learning from the model for different settings of the motor noise and exploration variability. This shows that performance tends to decrease with motor noise and intermediate values of exploration variability lead to the greatest reward. Therefore for a given level of motor noise there is an optimal level of exploration variability that will lead to maximal adaptation. Achieving a good level of adaptation thus requires a balance between exploration and motor noise.

Examining the fits across the subjects ( Fig. 7 B parameters with 95% confidence intervals) shows that there is wide variation of the parameters but that they tend to cluster by group. In addition there is variability in the per cent learning in the late perturbation period across subjects (percentage learning is shown as fill of the circular symbols in Fig. 7 B). The reason for this is that the model is probabilistic in nature and therefore even with identical parameters for exploration variability and motor noise the actual time series of reaches and adaptation will vary each time the model is run. For example, sometimes the model will be lucky and draw random samples of motor noise with less variability and sometimes the model will be unlucky and draw samples with more variability. We examined this by looking at the distribution of adaptation expected from the model when we performed many thousands of simulations and then determined where a participant’s actual adaptation lies within this distribution. Across all 34 participants we find on average that participants’ adaptation was ranked at 58 (where 1 is the model’s best and 100 the model’s worst performance) and that this rank was not significantly different from the expected value of 50 ( *P =* 0.11). Importantly, even though there was variation in performance predicted by the model, the way in which a participant changed their reach angle as a function of current reach angle and reward (or lack thereof) allowed us to extract model parameters with reasonably narrow confidence limits.

Analysis of mean parameter values yielded significant differences between groups. One-way ANOVA of motor noise revealed a main effect of group [σ _m_ ; *F* (2,31) = 7.6, *P <* 0.01; Brown-Forsythe, *F* (2,19.9) = 7.2, *P <* 0.01; Fig. 7 C] that resulted from significantly greater motor noise in the cerebellar group compared to both young ( *P <* 0.05) and age-matched controls ( *P <* 0.01). One-way ANOVA of exploration variability also revealed an effect of group [σ _e_ ; *F* (2,31) = 6.4, *P <* 0.01; Brown-Forsythe, *F* (2,15.2) = 5.7, *P <* 0.05, Fig. 7 C], which resulted from significantly greater exploration in young controls compared to both the cerebellar group ( *P <* 0.05) and older controls ( *P <* 0.01). Importantly, one-way ANOVA of the proportion of rewarded trials over the perturbation phase of the experiment revealed a main effect of group [ *F* (2,31) = 8.232, *P <* 0.01] where the reward rate was significantly lower for the cerebellar group (0.57 ± 0.04) than both the older (0.73 ± 0.04) and young control (0.76 ± 0.03) groups (both *P <* .01).

Although we have phrased the model as motor noise and exploration variability, a mathematically similar way of expressing this is that there is a total variability in a participant’s reach direction and that he or she is only aware of a proportion of this variability and corrects for this proportion when rewarded (see ‘Materials and methods’ section). We replot the parameter estimates as total variance and the proportion corrected for in Fig. 7 D. One-way ANOVA of total variance revealed a main effect of group [ *F* (2,31) = 9.9, *P <* 0.001; Brown-Forsythe, *F* (2,21.1724) = 10.0, *P <* 0.01] where both cerebellar ( *P <* 0.001) and young control ( *P <* 0.05) groups showed significantly greater variability than the older control group, but were not different from each other. Analysis of rho showed a significant main effect of group [ *F* (2,31) = 4.8, *P <* 0.05; Brown-Forsythe, *F* (2,11.2) = 4.1, *P <* 0.05] where the cerebellar group was aware of a smaller proportion of their total variability compared with both older and young controls. This comparison was significant between the cerebellar and young control group ( *P <* 0.05).

## Discussion

We examined the learning and retention of a visuomotor rotation using error-based and reinforcement feedback, and whether these mechanisms depend on the integrity of cerebellar function. Reinforcement schedules produced better retention compared with error-based learning. Moreover, using a closed-loop reinforcement schedule, where the reward was contingent on prior performance, produced rapid learning. Cerebellar patients could learn under the closed-loop reinforcement schedule and retained much more of the learned reaching pattern compared to when they performed error-based learning. However, cerebellar patients varied in their learning ability in the reinforcement condition, with some showing only partial learning of the rotation. We developed a computational model of the reinforcement condition and found that learning was dependent on the balance between motor noise and exploration variability, with the patient group having greater motor noise and hence learning less. Our results suggest that cerebellar damage may indirectly impair reinforcement learning by increasing motor noise, but does not interfere with the reinforcement mechanism itself.

We based the open-loop task on prior work showing binary reinforcement could drive visuomotor learning in controls ( Izawa and Shadmehr, 2011 ). However, in open-loop paradigms, subjects sometimes lag behind the perturbation to such an extent that they end up receiving no reward and no longer adjust their reaches or explore sufficiently to reacquire the target zone. We, therefore, included a closed-loop condition to mitigate this problem and to ensure that the reward schedule was set as close to 50% in the early period of learning. This was done by rewarding any reach that exceeded the average of the last 10 reaches in the desired direction (i.e. countering the rotation). This closed-loop paradigm led to more rapid learning than the open-loop reinforcement paradigm, with similar final levels and retention across our subjects.

While we designed our study to assess reinforcement and error-based learning in isolation, in general these mechanisms will work together in real-world situations. In the reinforcement task participants clearly do not have access to error information, whereas in the error-based task they have both error and task success (reinforcement) information. Nevertheless, our results show clear differences between the two paradigms, which suggests that we are largely studying distinct processes—one that is driven primarily by error that is not well retained and another clearly driven by reinforcement that is well retained. This is consistent with previous studies examining interactions between the two learning mechanisms, showing enhanced retention of an error-based learning process when reinforcement is also provided ( Shmuelof *et al.* , 2012 ; Galea *et al.* , 2015 ). In addition, combining error-based and reinforcement learning has been shown to speed up learning ( Nikooyan and Ahmed 2015 ).

Cerebellar patients have known deficits in error-based learning ( Martin *et al.* , 1996 ; Maschke *et al.* , 2004 ; Richter *et al.* , 2004 ; Morton and Bastian, 2006 ). Yet, the patients in our study were able to follow the gradual perturbation in the error-based condition. Analysis of their reach trajectories suggests that this ability relied on the patients using online visual feedback of the cursor to steer their reaching movements toward the target. As reach angles were calculated using the endpoint of each movement (this was necessary to compare the error-based task to the reinforcement tasks where reaches were rewarded based on movement endpoint), this feedback-dependent compensation made them appear to be learning. However, when visual feedback was removed to assess retention, the ability to correct the movement online was immediately lost (i.e. no retention). Thus, cerebellar patients did not truly adapt to the visuomotor rotation in the error-based task. Consistent with this, is that cerebellar patients cannot follow a gradual rotation when only endpoint cursor feedback is provided ( Schlerf *et al.* , 2013 ).

Reinforcement learning has been posited as a spared mechanism of motor learning following cerebellar damage. Consistent with this, Izawa *et al.* (2012) showed that cerebellar subjects could counter a gradual visuomotor rotation and generalize the new reach pattern to other targets when they learned with concurrent online visual cursor feedback and binary reinforcement feedback. They suggested that the cerebellar patients were relying on a reinforcement mechanism because they did not change the perceived location of their hand in a proprioceptive recalibration test, whereas control subjects did. Such proprioceptive recalibration is thought to be a hallmark of error-based adaptation ( Synofzik *et al.* , 2008 ). Here we specifically show that cerebellar patients can use binary reinforcement feedback alone to alter their reaching movements, although some patients were able to learn more than others. All patients, regardless of how much they learned, showed almost complete retention in the reinforcement task. This is in in stark contrast to the same patients showing a complete lack of retention in the error-based paradigm.

The failure of some cerebellar patients to learn in the reinforcement task could be due to either deficits in the reinforcement learning mechanism itself, or deficits in other mechanisms which might limit the usefulness of reinforcement learning (or a combination of the two). Cerebellar damage causes reaching ataxia, indicated by curved and variable reaching movements ( Bastian *et al.* , 1996 ). Patients with cerebellar damage also have proprioceptive impairments during active movements that are consistent with disrupted movement prediction ( Bhanpuri *et al.* , 2013 ). Together these studies suggest that cerebellar damage increases motor noise and/or reduces the sensitivity with which they can locate their own arm. In other words, this leads to variability in reaching that cannot be estimated by the brain. We suspected that such variability (which we consider a form of motor noise) might interfere with reinforcement learning.

To test this hypothesis we used a simple model of the reinforcement task in which each subject was characterized by two sources of variability—one that they were unaware of, which we call motor noise, and one that they were aware of, which we term exploration variability. We chose to use a simpler model than the one used by Izawa and Shadmehr (2011) , in which reward learning was based on a temporal difference learning algorithm. This algorithm requires specification of a range of parameters *a priori* (e.g. motor noise, discount rates, motor costs). In the temporal difference rule the current reward is compared to the expected reward to drive learning. However, due to the closed-loop nature of our paradigm, which set the perturbation so that the expected reward rate was always close to 50%, getting a reward was in general better than expected and failing to get a reward worse than expected. Therefore, we simplified the rule to update the motor command only for success and maintained the motor command for failure. This allowed us to avoid setting any parameters *a priori* and we fit two parameters to characterize subjects’ learning. Moreover, rather than fit squared error we were able to use a full probabilistic model using maximum likelihood, which allowed us to test whether our model was adequate to explain the subjects’ data. The model provided a good fit for the vast majority of subjects and showed that the patients’ had increased motor noise, but similar exploration variability compared to the matched controls. In other words, the majority of variability contributing to cerebellar patients’ behaviour could not be used by the motor system for learning. When reinforcement was received on successful trials, updating of internal estimates of the correct action (i.e. reach angle) was impaired—estimates could only be updated based on a small proportion of the movement variability (corresponding to the exploration component) resulting in less learning. The younger group had similar motor noise to the older control group but had higher exploration variability, which led to improved learning.

Previous work has noted that increased motor variability in the rewarded dimension of a reinforcement learning task is associated with more successful performance ( Wu *et al.* , 2014 ). These results suggested that behavioural variability might be a deliberate output of the motor system that is necessary during learning to explore the task space, find the optimal response and yield maximal reward. Our results are in general agreement with these findings as in a reinforcement learning task, exploration variability is essential. In general, for reinforcement learning the optimal amount of exploration variability will depend on the level of motor noise. Therefore, for a fixed level of motor noise subjects should ideally learn to set their exploration variability so as to have maximum adaptation. Although we have phrased the model as exploration variability and motor noise ( Fig. 7 C), a mathematically similar way of expressing this is that there is a total variability in a participant’s reach direction, but he or she is only aware of a proportion of this variability or only corrects for a proportion when rewarded ( Fig. 7 D). Under this interpretation of the model, the cerebellar subjects have higher overall variability and correct for less of this variability when they are successful.

In summary, we have shown that cerebellar patients are able to use binary reinforcement feedback alone to learn and retain a visuomotor reaching task. However, their motor noise interferes with this process. Future work is needed to determine if motor noise can be reduced or if increasing exploration variability can benefit these patients.
